# Anti-AQP4 autoantibodies promote ATP release from astrocytes and induce mechanical pain in rats

**DOI:** 10.1186/s12974-021-02232-w

**Published:** 2021-08-21

**Authors:** Teruyuki Ishikura, Makoto Kinoshita, Mikito Shimizu, Yoshiaki Yasumizu, Daisuke Motooka, Daisuke Okuzaki, Kazuya Yamashita, Hisashi Murata, Shohei Beppu, Toru Koda, Satoru Tada, Naoyuki Shiraishi, Yasuko Sugiyama, Katsuichi Miyamoto, Susumu Kusunoki, Tomoyuki Sugimoto, Atsushi Kumanogoh, Tatsusada Okuno, Hideki Mochizuki

**Affiliations:** 1 Department of Neurology, Osaka University Graduate School of Medicine, 2-2 Yamadaoka, Suita, Osaka, 565-0871 Japan; 2 Genome Information Research Center, Research Institute for Microbial Diseases, Osaka University, Suita, Osaka, Japan; 3 Department of Neurology, Kindai University Faculty of Medicine, Sayama, Osaka, Japan; 4 Graduate School of Data Science, Shiga University, Hikone, Shiga Japan; 5 Department of Respiratory Medicine and Clinical Immunology, Graduate School of Medicine, Osaka University, Suita, Osaka, Japan

**Keywords:** NMOSD, Pain, ATP, IL-1β

## Abstract

**Background:**

Intractable neuropathic pain is a common symptom of neuromyelitis optica spectrum disorder (NMOSD). However, the underlying mechanism of NMOSD pain remains to be elucidated. In this study, we focused on ATP, which is one of the damage-associated molecular patterns, and also a well-recognized molecule involved in peripheral neuropathic pain.

**Methods:**

We assessed the development of pain symptoms by injecting anti-AQP4 recombinant autoantibodies (rAQP4 IgG) into rat spinal cords. We incubated HEK293 cells expressing AQP4 (HEK-AQP4) and rat astrocytes with rAQP4 IgG and assessed the level of ATP in the supernatant. We performed transcriptome analysis of the spinal cords injected with rAQP4 IgG. Pharmacological inhibition was also applied to investigate the involvement of ATP in the development of neuropathic pain in our rat model. The ATP concentration within the cerebrospinal fluid was examined in patients with NMOSD and other neurological diseases.

**Results:**

Development of mechanical allodynia was confirmed in rAQP4 IgG–treated rats. AQP4-Ab–mediated extracellular ATP release from astrocytes was observed in vitro, and pharmacological inhibition of ATP receptor reversed mechanical allodynia in the rAQP4 IgG–treated rats. Furthermore, transcriptome analysis revealed elevation of gene expressions related to several ATP receptors including *P2rx4* and *IL1B* in the spinal cord of rAQP4 IgG–treated rats. In patients, CSF ATP concentration was significantly higher in the acute and remission phase of NMOSD than in multiple sclerosis or other neurological disorders.

**Conclusion:**

Anti-AQP4 antibody was shown to induce the release of extracellular ATP from astrocytes. The ATP-mediated development of mechanical allodynia was also suggested in rats treated with anti-AQP4 antibody. Our study indicates the pivotal role of ATP in the pain mechanism of NMOSD.

**Supplementary Information:**

The online version contains supplementary material available at 10.1186/s12974-021-02232-w.

## Background

Neuromyelitis optica spectrum disorder (NMOSD) is an autoimmune inflammatory disease of the central nervous system that presents with optic neuritis or longitudinally extensive myelitis and is associated with various neurological symptoms [1]. Approximately 80% of patients with NMOSD experience intractable pain, and their quality of life is severely affected [2]. Current therapeutics for NMOSD pain, including antiepileptic agents, anti-spasticity medications, and opioids, provide insufficient relief of the symptoms [3]. A few studies reported pain development in a NMOSD animal model [4]. However, the detailed analysis of the mechanism related to NMOSD pain has not been performed in the past literature [2]. Consequently, the detailed pathogenesis of NMOSD pain remains to be elucidated.

Anti-aquaporin4 autoantibody (AQP4-Ab) plays a pathogenic role in NMOSD [5–7]. We showed previously that mitochondrial DNA (mtDNA), a damage-associated molecular pattern (DAMP), is released by dying astrocytes in an AQP4-Ab–dependent manner and further augments inflammation via innate immune receptors [8]. Damage-associated molecular patterns (DAMPs), such as high mobility group box-1 (HMGB1), mtDNA and ATP, are known to accelerate innate immune responses [9]. Moreover, the pivotal roles of ATP and purinergic receptors have been demonstrated in a peripheral neuropathic pain model [10].

In this study, we sought to clarify whether ATP is involved in the pathogenesis of NMOSD pain. ATP is previously shown to activate microglia and further promote secretion of IL-1β, which result in the activation of neuronal cells in a peripheral neuropathic pain model [11]. However, there has been no previous report investigating the involvement of ATP in NMOSD.

## Methods

### Patient information and sample collection

CSF was obtained from patients with AQP4-Ab–positive NMOSD in the acute (*n* = 17) or remission phase (*n* = 18), MS in the acute (*n* = 15) or remission phase (*n* = 6), or other neurological diseases (ONDs, *n* = 56). All NMOSD subjects were diagnosed according to the 2015 NMOSD diagnostic criteria, and all MS patients fulfilled the 2010 McDonald criteria. ONDs included Guillain-Barré syndrome (GBS) (*n* = 6), amyotrophic lateral sclerosis (ALS) (*n* = 27), Parkinson disease (PD) (*n* = 6), idiopathic normal pressure hydrocephalus (iNPH) (*n* = 5), cervical spondylosis (*n* = 5), and somatic symptom disorders (*n* = 7). The acute phase of NMOSD was defined as a sudden appearance of new neurological symptoms, and CSF during the acute phase was collected before or within 24 h from the start of treatment (high-dose intravenous methylprednisolone or plasmapheresis). CSF in the remission phase was collected after symptoms resolved following treatment for the acute phase. Informed consent was obtained from each patient. This study was approved by the ethics committee of Osaka University Hospital (permit number 12091-6).

### Surgical procedures

Female Lewis rats (age, 8 weeks; body weight, 200–250 g) purchased from the Charles River Laboratories Japan (Yokohama, Japan) were anesthetized with a mixture of Dormicam (4 mg/kg), Vetorphale (5 mg/kg), and Domitor (4 mg/kg), and laminectomy was performed at the thoracic 10 (Th10) vertebral level, where the spinal cord was exposed. A microsyringe (75RN, Hamilton, Reno, NV, USA) was inserted 1.5 mm at Th10 and used to infuse patient-derived AQP4 recombinant antibody [12] or control IgG [Human IgG1 lambda, Nordic-MUbio BV, Susteren, Netherlands] (20 μg), 1 μL of normal human serum, and indocyanine green (Tokyo Chemical Industry, Tokyo, Japan). Total volume was 4 μL. Rats in the TNP-ATP–treated group received a mixture of 1 μg TNP-ATP (SM0740, Sigma-Aldrich, St. Louis, MO, USA). All experimental procedures were performed according to the protocols approved by the Animal Care and Use Committee of Osaka University School of Medicine and the US Public Health Service Policy on Humane Care and Use of Laboratory Animals.

### Immunohistochemistry

HEK293 cells or HEK293 cells expressing AQP4 (HEK-AQP4) were fixed in 4% paraformaldehyde for 15 min. After incubation with 1% BSA in PBS for 30 min at room temperature, cells were incubated with anti-AQP4 IgG FITC (1:50; Santa Cruz Biotechnology; Santa Cruz, CA, USA) overnight at 4 °C. Rats were euthanized by terminal anesthesia. Spinal cords were embedded in Tissue Tek OTC compound 4583 (Sakura Finetech, Tokyo, Japan) and snap-frozen. Twenty micrometer-thick frozen tissue sections were fixed in 4% paraformaldehyde for 15 min. To stain GFAP and AQP4, after incubation with 1% BSA in PBS for 30 min at room temperature, the sections were incubated with anti-AQP4 (1:50; SAB5200112, Sigma-Aldrich, St. Louis, MO, USA) overnight at 4 °C. The sections were incubated with goat anti-rabbit IgG Alexa Fluor 594 (1:200; Abcam, Cambridge, UK). Then, the section was incubated with anti-GFAP Alexa Fluor 488 conjugate (1:50; 3655 Cell Signaling Technology, Beverly, MA, USA). Images were captured on a BZ-X710 microscope (Keyence, Osaka, Japan).

### Behavioral studies

To assess mechanical sensitivity, calibrated von Frey filaments (Aesthesio, DanMic Global, San Jose, CA, USA) were applied to the plantar surfaces of the hindpaws of NMOSD and control rats. The rats were placed in a plastic cage with a wire mesh bottom that allowed access to their paws. Behavioral acclimatization was allowed for 1 h until cage exploration and grooming activities ceased. The area tested was the mid-plantar hindpaw. Series of von Frey monofilaments with different pressure intensities (from 1.0 to 26 g) were applied. The threshold of pain was determined as the lowest pressure filament that induced avoiding response in five out of ten applications.

### Cell cultures

HEK293 cells transfected with or without M23-human AQP4 expression plasmid (GeneCopoeia, Rockville, MD, USA) were cultured in Dulbecco’s modified Eagle’s medium (DMEM) containing 10% fetal bovine serum and 1% penicillin–streptomycin. The M23-human AQP4 plasmid was transfected into HEK293 cells by using FuGENE6 Transfection Reagent (Roche Diagnostics, Indianapolis, IN, USA) following the manufacturer’s instructions. Clones of stably transfected cells were selected by 1.0 mg/ml G418. The selected clones were confirmed to show high expression of AQP4 by western blot analysis and immunocytochemistry. Primary astrocytes were obtained from Sprague–Dawley rats as previously described [13] according to the protocols approved by the Animal Care and Use Committee of Osaka University School of Medicine and the US Public Health Service Policy on Humane Care and Use of Laboratory Animals. Immunohistochemical analysis confirmed that more than 95% of collected cell population consisted of GFAP-positive astrocytes.

### Quantification of ATP

HEK293 cells were seeded in 24-well culture plates and stimulated with DMEM with or without AQP4 recombinant antibody [12] containing 0, 5, or 10% normal human serum at 37 °C. Culture supernatants were collected 2 h after antibody stimulation and centrifuged at 2000×*g* at 4 °C for 10 min. Rat primary astrocytes were seeded in 24-well culture plates and stimulated with DMEM with or without AQP4 recombinant antibody containing 10% NHS at 37 °C. Culture supernatants were collected 2 h after antibody stimulation and centrifuged at 2000×*g* at 4 °C for 10 min. Supernatants were analyzed using the ATP Bioluminescence Assay Kit HS II (Roche, Basel, Switzerland). The standard samples and control groups were added to the corresponding wells. CSF samples were centrifuged at 2000×*g* at 4 °C for 10 min. ATP levels in CSF samples of patients were measured using the ATPlite luminescence assay system (PerkinElmer, Waltham, MA, USA). In this assay, the emitted light produced by the reaction of ATP with added luciferase and D-luciferin is proportional to the concentration of ATP. In a 96-well white plate, 50 μL of the reconstituted reagent was added to each well, which contained 100 μL of CSF supernatant centrifuged at 2000×*g* at 4 °C for 10 min and equilibrated at room temperature. Luminescence was measured using a Centro XS3 LB960 luminescent microplate reader (Berthold Technologies, Bad Wildbad, Germany).

### Transcriptome analysis

For transcriptome analysis of spinal cord tissue, rAQP4 IgG– or control IgG–treated rats were transcardially perfused with ice-cold PBS under anesthesia with i.p. injection of a mixture of Dormicam (4 mg/kg), Vetorphale (5 mg/kg), and Domitor (4 mg/kg). Spinal cords (*n* = 3 each group) were cut out at the Th10 level, and total RNA was prepared using ISOGEN II (Nippongene, Toyama, Japan). RNA libraries were prepared for sequencing using the TruSeq Stranded mRNA Library Prep kit (Illumina, San Diego, CA, USA). Sequencing was performed on the Illumina HiSeq 2500 platform in a 75-bp single-end mode. Sequenced reads were mapped to the rat reference genome sequence (rn6) using TopHat ver. 2.1.1 in combination with Bowtie2 ver. 2.2.3. The raw counts were calculated using Cufflinks ver. 2.2.1. Transcriptome analysis was performed using either iDEP.91 [14], the STRING database [15], or Enrichr [16]. For principal component analysis (PCA), normalization and scaling of raw read counts were processed by the EdgeR package of iDEP.91 (filtered with 0.5 counts per million); among the six samples, 12,453 genes passed the filter. Differentially expressed genes (DEGs) were assessed by DESeq2 (false discovery rate cutoff < 0.1, fold change > 2) and visually depicted using the STRING database. Heat map of purinergic receptors was calculated with iDEP.91 by normalizing samples against the standard deviation. Using Enrichr-based WikiPathways analysis, gene set enrichment analysis was performed with upregulated DEGs of NMOSD rats that met the criterion |log_2_ (fold-change)| > 1. Raw data from this analysis were submitted to Gene Expression Omnibus (GEO) under accession number GSE150598.

### Quantitative PCR

Total RNA of the spinal cords was obtained as described in the “Transcriptome analysis” section. Sixty nanograms RNA extracted according to the instruction protocol was reverse transcribed with SuperScript VILO (Thermo Fisher Scientific, San Jose, CA, USA) as follows: 25 °C for 10 min, 42 °C for 60 min, and 85 °C for 5 min. cDNA corresponding to 1ng RNA was measured with q-PCR. The q-PCR was performed using TaqMan probes according to the manufacturer’s protocol (Applied Biosystems, Foster City, CA, USA), and the reactions were performed using the Applied Biosystems 7900HT (Applied Biosystems, Foster City, CA, USA). The following TaqMan primers were used: Rn00580432_m1 (interleukin 1 beta; *IL1B*) and Rn00667869_m1 (Actin beta; *Actb*). The resultant data were analyzed according to the comparative cycle threshold (Ct) method (ΔΔCt), which was normalized to the expression of *Actb* reference genes.

### Statistics

Data are shown as means ± SEM. Data assessing allodynia and HEK-AQP4 ATP assay were analyzed by linear mixed regression analysis for factorial repeated measures ANOVA model with random-effects of one within-subjects (the same rat’s paired right and left data), with post hoc Bonferroni correction. Rat astrocyte ATP assay and quantitative polymerase chain reaction analysis were analyzed by Student’s *t* test. CSF ATP levels of different groups were analyzed by Welch’s *T* test. Statistical significance was defined as *P* < 0.05. Asterisks in figures denote *P* values as follows: *P < 0.05 and **P < 0.01.

## Results

### Administration of rAQP4 IgG to rats induces the development of mechanical pain

**Fig. 1:**
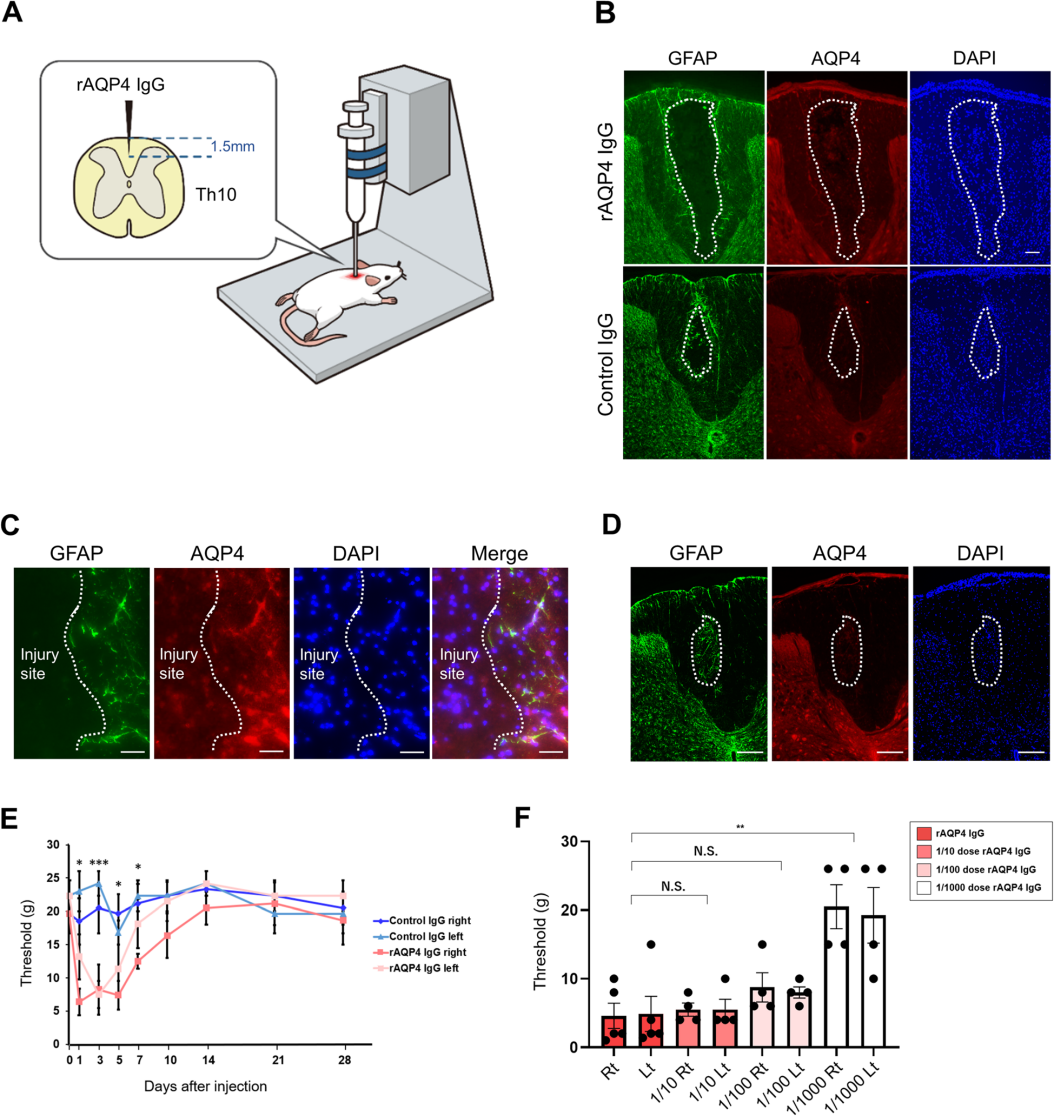
Intraspinal injection of rAQP4 IgG induces mechanical allodynia. **A** Schema of experimental procedures. **B** Representative images of GFAP, AQP4, and DAPI staining in the spinal cords of rats 3 days after receiving rAQP4 IgG or control IgG. Scale bar = 200 μm. **C** High magnification images of the injury site in rAQP4 IgG–treated group. Astrogliosis is observed in peripheral lesion of the injury site. Scale bar = 100μm. **D** GFAP, AQP4, and DAPI staining at the injection site of the spinal cord in rAQP4 IgG–treated group 5 days after the injection. Scale bar = 200 μm. **E** Time course of thresholds of mechanical allodynia. Thresholds of mechanical allodynia assessed 0 (pre-injection), 1, 3, 5, 7, 10, 14, 21, and 28 days after intraspinal injection of either rAQP4 IgG (*n* = 6) or control IgG (*n* = 6). Data are expressed as means ± SEM and were analyzed by factorial repeated measures ANOVA with three between-subjects factors [group: rAQP4 and control; day 1, 3, 5, 7, 10, 14, 21, and 28; RL: right (Rt) and left (Lt)] and one within-subjects factor [the same rat’s paired Rt and Lt data]. The main effect of group (*P* = 0.018) and the interactions of group times day (*P* < 0.001) and group times RL (*P* = 0.045) were statistically significant. For the visualization, the asterisks indicate two-group comparison at each day. **F** Thresholds of mechanical allodynia assessed 3 days after intraspinal injection of either undiluted rAQP4 (*n* = 5), 1/10 dose rAQP4 IgG (*n* = 4), 1/100 dose rAQP4 IgG (*n* = 4), or 1/1000 dose rAQP4 IgG (*n* = 4). Data are expressed as means ± SEM and analyzed by two-way repeated measures ANOVA with one within-subjects factor [the same rat’s paired right (Rt) and left (Lt) data]. ***P < 0.001, **P < 0.01, *P < 0.05. N.S., not significant

To determine whether AQP4 antibodies induce pain symptoms in rats, we used recombinant monoclonal AQP4 antibody (rAQP4 IgG) from NMOSD patient plasmablasts, which was previously established in our laboratory [12]. The rAQP4 IgG recognizes extracellular domain of AQP4 and have high capacity to induce complement-dependent cytotoxicity [12]. The rAQP4 IgG was shown to directly bind to astrocytes and the target antigen was confirmed to be AQP4 (Fig S1). Patient-derived rAQP4 IgG was stereotaxically injected into rat spinal cords at the T10 vertebra (Fig. 1A). The injection sites of both rAQP4 antibody-treated and control IgG–treated groups were unavoidably accompanied by injurious pathological changes caused by needle insertion and sample infusion. This being said, rAQP4 antibody-treated group showed larger lesion formation in certain sections (Fig. 1B). Higher magnification images demonstrated the presence of astrogliosis in peripheral lesion of injury site (Fig. 1C). Additionally, astrogliosis observed in the spinal cords was shown to persist also 5 days after the injection (Fig. 1D). The rats were tested for mechanical and thermal paw withdrawal responses. Relative to the control group, rats receiving rAQP4 IgG exhibited a lower threshold for mechanical pain in both hind limbs. The mechanical pain was observed one day after rAQP4 IgG injection and peaked at day three and lasted for 1 week (Fig. 1E). By contrast, thermal paw responses did not differ significantly between the two groups (Fig S2). No apparent motor deficit was observed in both groups. We also performed a dose dependent trial. The results indicated that 1/1000 dose rAQP4 IgG was not enough to induce pain symptoms in our rat model (Fig. 1F). These results demonstrate that patient-derived rAQP4 IgG is capable of inducing mechanical allodynia in rats. To confirm the universal property of anti-AQP4 antibody to induce pain symptoms in rats, rats were injected with sera derived from either anti-AQP4 antibody–positive patients or healthy control. The induction of mechanical pain was observed exclusively in rats injected with anti-AQP4 antibody–positive NMOSD patient serum (Fig S3).

### rAQP4 IgG promotes extracellular ATP release

**Fig. 2:**
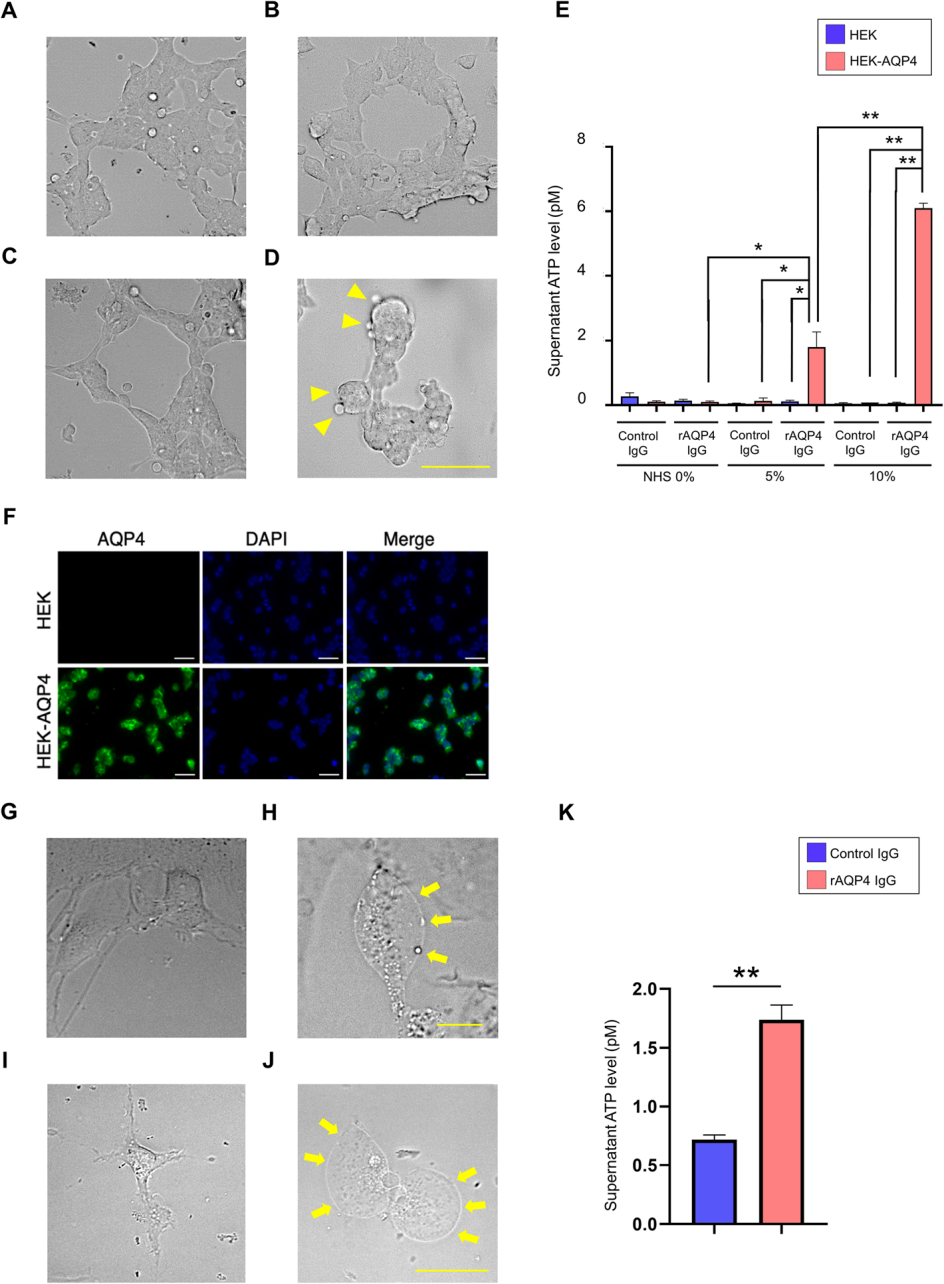
rAQP4 IgG promotes extracellular ATP release. HEK293 cells (**A**, **B**) or HEK293 cells expressing AQP4 (**C**, **D**) were stimulated with control IgG (**A**, **C**) or rAQP4 IgG (**B**, **D**) in the presence of 5% normal human serum (NHS). Only HEK-AQP4 incubated with rAQP4 IgG underwent a balloon-like morphological change (arrowheads) (**D**). Scale bar = 100 μm. (**E**) Extracellular ATP level in the culture medium. HEK293 cells (HEK) or HEK293 cells expressing AQP4 (HEK-AQP4) were incubated with either control IgG or rAQP4 IgG in the presence of various concentrations of complement. Data are expressed as means ± SEM and were analyzed by factorial ANOVA with three between-subjects factors [NHS, 0%, 5%, or 10%; group: rAQP4 IgG or control IgG; HEK-AQP4, HEK or HEK-AQP4]. The main effects of NHS (*P* < 0.001), group (*P* < 0.001), and HEK-AQP4 (*P* < 0.001) and the interactions of NHS times group (*P* < 0.001), NHS times HEK (*P* < 0.001), and group times HEK (*P* < 0.001) were statistically significant. For the visualization, the corresponding two-group comparisons were indicated by the asterisks, *P < 0.05 and **P < 0.01. **F** HEK and HEK-AQP4 were immunostained with anti-AQP4 antibody. HEK-AQP4 were confirmed to express AQP4 antigen. Scale bar = 25 μm. Rat astrocytes were stimulated with control IgG (**G**, **I**) or rAQP4 IgG (**H**, **J**) in the presence of 10% NHS (**G**, **H**) or 2% rabbit complement (**I**, **J**). Only rat astrocytes incubated with rAQP4 IgG exhibited a balloon-like morphological change (arrows). **G**, **H** Scale bar = 50 μm. **I**, **J** Scale bar = 100μm. **K** Rat astrocytes were stimulated with human IgG or rAQP4 IgG in the presence of 10% NHS. Data are expressed as means ± SEM and were analyzed by Student’s *t* test and **P < 0.01

Among the molecules reported to play pivotal roles in peripheral neuropathic pain, ATP is an essential factor that also functions as a DAMP in immune-mediated cellular damage. Hence, to determine whether rAQP4 IgG promotes ATP release from cells expressing AQP4, we incubated HEK-AQP4 with patient-derived rAQP4 IgG. After 1 h of incubation, the rAQP4 IgG–treated cells exhibited a balloon-like morphology (Fig. 2A–D). Concomitant with the morphological alteration, the level of ATP in the supernatant was elevated in the rAQP4 IgG–treated cells. Importantly, the amount of ATP released was higher in the presence of 10% normal human serum addition than in the presence of 5% serum (Fig. 2E). The ATP level was remarkably reduced when heat deactivated human serum was applied instead of normal human serum (Fig S4). HEK-AQP4 were confirmed to express AQP4 antigen by immunocytochemistry (Fig. 2F). These results indicate that in the presence of complement, rAQP4 IgG induces a balloon-like morphological change in HEK-AQP4 cells, followed by extracellular release of ATP. Similarly, we incubated rat astrocytes with either control IgG or rAQP4 IgG in the presence of normal human serum (Fig. 2G, H). After 1 h of incubation, the rAQP4 IgG–treated astrocytes exhibited balloon-like morphology. The treatment of rat astrocytes with control IgG or rAQP4 IgG in the presence of 2% rabbit complement also demonstrated balloon-like morphological change exclusively in rAQP4 IgG–treated group (Fig. 2I, J). Moreover, rAQP4 IgG also induces extracellular ATP release from rat primary astrocytes (Fig. 2K).

### Inhibition of ATP receptors prevents mechanical pain of rAQP4 IgG–treated rats

**Fig. 3:**
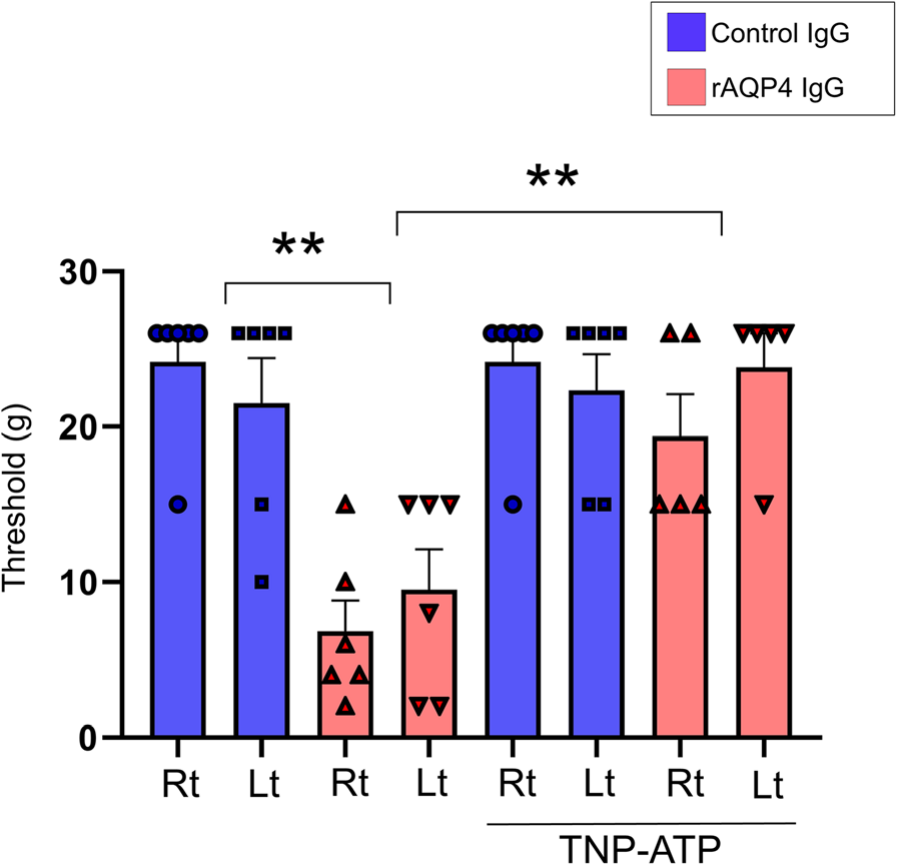
TNP-ATP reverses mechanical allodynia. Thresholds for mechanical allodynia assessed in rats receiving either control IgG or rAQP4 IgG, with or without Intraspinal administration of TNP-ATP (*n* = 5–6 each group). Data are expressed as means ± SEM and were analyzed by factorial repeated measures ANOVA with two between-subjects factors [group: rAQP4 IgG and control IgG; TNP-ATP: with or without TNP-ATP] and one within-subjects factor [the same rat’s paired right (Rt) and left (Lt) data]. The main effects of Group (*P* < 0.001) and TNP-ATP (*P* = 0.003) and the interaction of Group times TNP-ATP (*P* = 0.003) were statistically significant. For the visualization, the corresponding two-group comparisons were indicated by the asterisks, **P < 0.0.

Given that our in vitro study showed that rAQP4 IgG induced the extracellular release of ATP, we next assessed whether ATP receptor antagonist would prevent the development of neuropathic pain in the rAQP4 IgG–treated rats. To this end, we locally co-administered TNP-ATP (an antagonist of P2RX1, P2RX3, P2RX2/3, and P2RX4) when rats were injected in the spinal cord with either rAQP4 IgG or control IgG. Three days after injection, the rats were tested for mechanical paw withdrawal responses. In the rAQP4 IgG–treated group, TNP-ATP prevented mechanical pain symptoms, bringing the threshold of mechanical allodynia to a level comparable to that in the control IgG group (Fig. 3). These results indicated that ATP plays an important role in the pathogenesis of NMOSD pain.

### Transcriptome profiling of the spinal cord in rAQP4 IgG–treated rats

**Fig. 4:**
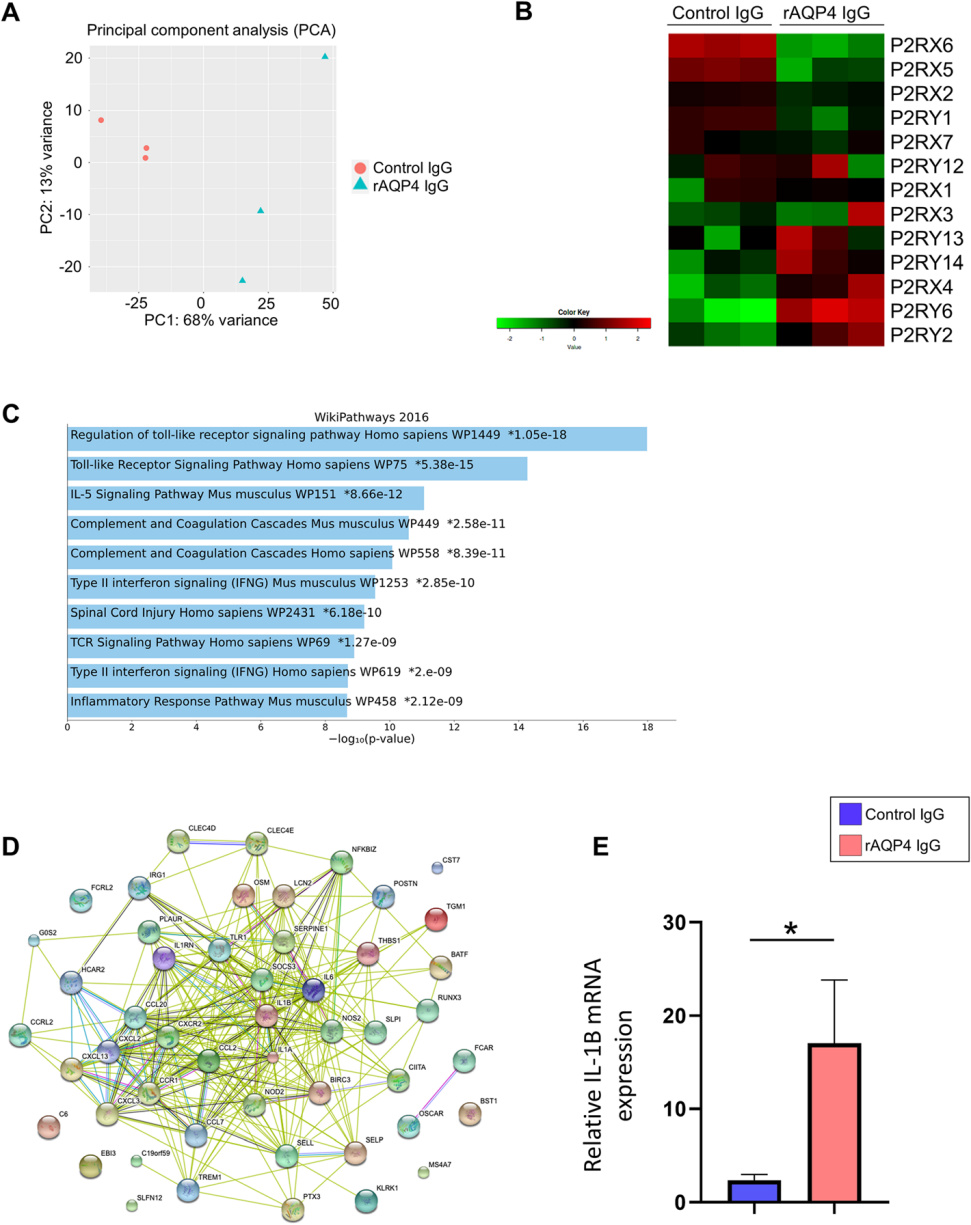
Transcriptome profiling of the NMOSD pain model reveals IL-1β is a hub of molecular events. **A** PCA of transcriptome data obtained from spinal cords of rats receiving either control IgG or rAQP4 IgG. **B** Heat map representing expression levels of purinergic receptors. **C** Gene set enrichment analysis of genes upregulated in the spinal cord in the NMOSD pain model. The bar chart shows the top 10 enriched terms in WikiPathways 2016, along with their corresponding *p*-values. Colored bars correspond to terms with significant *p*-values. An asterisk next to a *p*-value indicates the term also has a significant adjusted *p*-value. **D** The protein–protein interaction network of the top 50 upregulated differentially expressed genes in spinal cords of rats receiving rAQP4 IgG, ranked by fold change. The graph was generated using the STRING database. The nodes represent differential genes and the number of edges reflects known protein–protein associations. Differently colored edges indicate different types of evidence used in predicting interactions. Red line indicates the presence of fusion evidence. Green line indicates neighborhood evidence. Blue line indicates cooccurrence evidence. Purple line indicates experimental evidence. Yellow line indicates textmining evidence. Light blue line indicates database evidence. Black line indicates coexpression evidence. **E** Gene expression of *IL1B* in rat spinal cord of either rAQP4 IgG or control IgG–treated group. Data are expressed as means ± SEM, and were analyzed by Student’s *t*-test and *P < 0.05

To further clarify the molecular pathway in rats treated with rAQP4 IgG, we performed RNA sequence on spinal cords of rAQP4 IgG– or control IgG–treated rats 3 days after the antibody injection. Principal component analysis (PCA) revealed a clear difference in gene expression between the two groups (Fig. 4A). Among the differentially expressed genes, several ATP receptors including *P2rx4*, were elevated in rAQP4 IgG–treated rats, (fold change = 2.154, *p*-value = 0.0194). *P2rx7* was not significantly elevated in rAQP4 IgG–treated rats (fold change = 1.304, *p*-value = 0.0608) (Fig. 4B). Gene Ontology (GO) enrichment analysis revealed that the genes involved in Toll-like receptor signaling, chemokine signaling, IL-5 signaling, and complement cascades, all of which play important roles in human NMOSD pathology, were highly enriched in rAQP4 IgG–treated rats (Fig. 4C). Furthermore, a network analysis of known and putative protein–protein interactions in the NMOSD model revealed that the upregulated genes are connected, and that some of them are hub genes, e.g., *IL1B*, which is a core downstream molecule in pain hypersensitivity induced by peripheral nerve injury and highly expressed in early active inflammatory lesions in NMOSD patients [17] (Fig. 4D). Moreover, the fold change of *IL1B* was one of the highest among the genes which were upregulated in our model (fold change = 37.604, *p*-value = 0.0117) (Table. S1). The elevation of *IL1B* expression was also confirmed by quantitative polymerase chain reaction analysis (Fig. 4E). Additionally, gene expression of *iNOS* (fold change = 71.894, *p*-value = 0.00459), *IRF5* (fold change = 1.909, *p*-value = 0.011), and *IRF8* (fold change = 2.695, *p*-value = 0.00260) were also elevated in rAQP4 IgG–treated rats.

### Elevation of CSF ATP level is a unique feature of patients with NMOSD

**Fig. 5:**
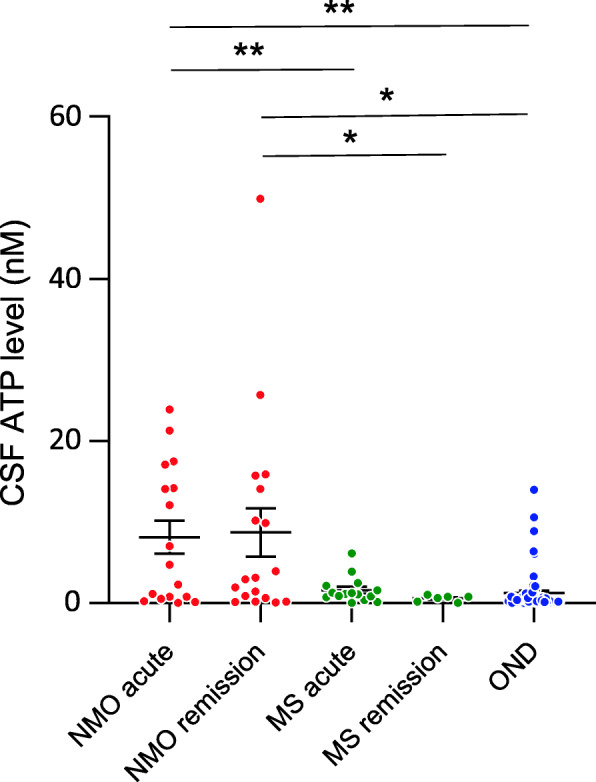
Elevation of CSF ATP in patients with NMOSD. CSF ATP levels among patients with NMOSD in acute or remission phase, MS in acute or remission phase, or other neurological diseases (OND). Data are expressed as means ± SEM and were analyzed by Welch’s *T* test. *P < 0.05 and **P < 0.01

To determine whether concentration is specifically elevated in cerebrospinal fluid (CSF) of NMOSD patients, we assessed the ATP level in the CSF of patients with NMOSD, multiple sclerosis (MS), or other neurological diseases (ONDs). CSF ATP levels were significantly higher in patients with NMOSD than in those with MS, in both the acute and remission phases. Similarly, CSF ATP levels were significantly higher in NMOSD patients than in OND patients. These results suggest that elevation of CSF ATP levels is a unique feature of patients with NMOSD (Fig. 5). We also measured IL-1β and IL-6 in CSF derived from NMOSD patients. However, both IL-1β and IL-6 concentration level were under the detection levels in majority of patients with NMOSD and MS (Fig S5).

## Discussion

In this study, we showed that AQP4-Ab mediates extracellular ATP release from astrocytes and that pharmacological inhibition of P2XR reverses mechanical allodynia in the rAQP4 IgG–treated rats. This is in line with the previous report which showed intrathecal administration of ATP produced mechanical allodynia, and co-administration of purigernic receptor antagonist completely prevented the induction of pain [18]. Furthermore, we found that the CSF ATP concentration was significantly higher in the acute and remission phase of NMOSD than in MS and other neurological disorders. Transcriptome analysis revealed elevated *IL1B* expression in rAQP4 IgG–treated rat spinal cord. IL-1β is known to be a downstream inflammatory cytokine produced by microglia via ATP-mediated signaling cascade and is reported to induce peripheral neuropathic pain [9].

Rodent models of peripheral neuropathic pain, like spared nerve injury (SNI), are well established in the pain research field [19]. However, few studies have developed pain models for inflammatory diseases of the CNS. Hillebrand et al. reported pain symptoms in a NMOSD animal model, which was established by intraperitoneally injecting monoclonal AQP4 specific antibody [4]. However, there were no previous reports that showed detailed mechanism of NMOSD pain development. We previously reported an NMOSD rat model generated by intraperitoneal injection of IgG derived from NMOSD patients [7]. Although these rats exhibited pathological changes characteristic to NMOSD patients, due to their severe clinical symptoms they were not suitable for accurately assessing the pain symptoms of NMOSD. Therefore, in this study, we chose to perform intraspinal injection of patient-derived monoclonal AQP4-Ab [12] so that lesion formation was restricted and the rats were spared from lower limb immobilization. As an experimental control, we prepared control IgG–treated rats following exactly the same surgical procedure as rAQP4 IgG–treated group. The injection sites of both rAQP4 antibody-treated and control IgG–treated groups were accompanied by injurious pathological changes. However, larger lesion formation was confirmed in rAQP4 antibody-treated group in certain sections, supporting the pathogenic effect of rAQP4 antibody. Whether the astrogliosis observed in our study represent the mechanically induced injurious pathological changes, or the chronological alteration as recently reported in NMOSD pathology remains to be elucidated in a future study [20]. These control IgG–treated rats also served as the control to examine the effect of surgical injury on the development of neuropathic pain.

In an animal model of peripheral neuropathic pain, SNI procedure is usually applied at either one of the bilateral nerves, inducing neuropathic pain only at ipsilateral hindpaw [19]. In this study, based on the fact that lesions of human NMOSD typically involve the central spinal cord [21], we injected rAQP4–IgG at midline portion of spinal cords and the rats developed mechanical allodynia at bilateral hindpaws. In peripheral neuropathic pain model, it is reported that different immune cells mediate mechanical hypersensitivity in male and female mice. And microglia are not required for mechanical pain in female mouse [22]. Clinically, about 80% of NMOSD patients are female, so we used female rats in this study [23]. The rAQP4 IgG–treated rats presented mechanical pain, but pain related to NMOSD patients is not limited to mechanical allodynia. Spontaneous painful sensations are also common in NMOSD. Thus, it would be more informative to establish a NMOSD pain animal model which represents the full spectrum of pain symptoms in future studies. In our study, injection of AQP4-Ab was performed at T10 vertebral level of spinal cord (L1-2 spinal cord level), which is a little distant from L4-5 spinal cord where the nociceptive signals from hind paws enter. These apparently discrepant phenomena can be explained by the previous observation that thoracic tissue damage can propagate to activate L4-5 spine microglia and induce pain symptoms [24]. Transcriptome analysis of spinal cords or rAQP4 IgG–treated rats revealed elevation of gene sets associated with NMOSD pathology, including chemokine signaling, IL-5 signaling, and complement cascades. AQP4-Abs attract granulocytes to lesion sites via chemokine signaling, and IL-5 promotes further tissue injury by activating eosinophils [25]. AQP4-Abs also induce complement-dependent cellular cytotoxicity [26]. These results confirmed that rAQP4 IgG–treated rat spinal cord reflects the characteristics of human NMOSD pathogenesis based on gene expression pattern.

CSF levels of HMGB1, a DAMP, are higher in NMOSD patients than in MS and ONDs patients [27]. In addition, the mtDNA level is specifically elevated in NMOSD CSF [8]. Cellular stress and necrosis are thought to be major contributors to DAMPs release. Thus, the higher CSF DAMPs levels in NMOSD presumably reflect the destructive nature of NMOSD pathology, which is mediated by AQP4-Ab–dependent astrocyte damage. We found that AQP4-Ab mediates extracellular ATP release from HEK293 cells expressing AQP4. ATP is an essential energy source for cells, but recent studies have revealed that it also acts as a DAMP and further influences cellular migration and pain induction. In peripheral neuropathy models, injured neurons release ATP in the dorsal horn of the spinal cord [28], which is detected by purinergic receptors expressed by microglia, thereby inducing pain. In another model, *C. albicans*–derived β-glucan promotes ATP release from keratinocytes and also elicits pain symptoms [29]. CSF level of ATP was higher in NMOSD patients than those of MS and ONDs patients. Therefore, we speculated that ATP released from dying astrocytes following complement-dependent necrosis functions as a chemical mediator to modulate NMOSD neuropathic pain. There was no correlation between visual analog scale (VAS) score and CSF ATP level among NMOSD patients in our cohort (data not shown). This might be reflecting the fact that the lesion location was variable among the NMOSD patients in our cohort, thus not necessarily involving the spinal cord lesions which are responsible for the development of pain symptoms in NMOSD [21]. It will be interesting to examine the correlation of VAS score and CSF ATP level in a larger cohort in future studies.

In this study, TNP-ATP, a P2RX4 antagonist, attenuated mechanical allodynia in the rAQP4 IgG–treated rats. Multiple reports have highlighted the importance of purinergic signaling and microglial activation in the pathogenesis of peripheral neuropathic pain. Nerve injury induces upregulation of transcriptional factors of IRF8 and IRF5, along with the purinergic receptor P2RX4, exclusively in spinal cord microglia [30, 31]. Furthermore, nerve injury–induced allodynia is abolished in *P2rx4*-knockout mice, and intrathecal injection of P2RX4-stimulated microglia can cause allodynia [32]. In addition, nerve injury induces upregulation of *P2rx7* in spinal cord microglia [33]. Interestingly, P2RX7 and P2RX4 form functional interactions through their physical association [34, 35]. Activation of P2RX4 and P2RX7 leads to microglial activation via p38 phosphorylation; increases the synthesis and release of various chemical mediators such as TNF-α, IL-1β, IL-6, BDNF, and PGE2; and facilitates the development of neuropathic pain [36]. In our rAQP4 IgG–treated rat spinal cord, the expression of *P2rx4*, but not *P2rx7,* was significantly elevated in rAQP4 IgG–treated rats. Moreover, the expression of *IL1B* was one of the highest in all the genes analyzed. The expression of *IL-6* was also elevated and that of *BDNF* was not elevated (data not shown).

Notably in regard to the role of IL-1β in pain development, P2RX4 regulates P2RX7-dependent IL-1β release [37]. Additionally, microglia are suggested to be the major source of IL-1β in NMOSD pathology [17]. In line with these observations, in this study we revealed that IL-1β constitutes one of the hubs of molecular events occurring in NMOSD rat models by transcriptome analysis. IL-1β acts on neurons to increase glutamate release [38]; in addition, *Il1r1*-knockout mice do not develop acute pain in a paclitaxel-induced model [39]. Therefore, it could be speculated that IL-1β secretion also plays key roles in NMOSD neuropathic pain.

One limitation of our study is that our rAQP4 IgG–treated rat presented pain only at the acute phase of the disease. Clinically, pain of NMOSD patients is most prominent in the chronic phase of the disease [2]. Thus, it would be important to establish the model which more precisely recapitulates the clinical pain syndrome of NMOSD in future studies. Moreover, it would be nice to show the elevation of ATP contents in CSF or spinal cords of rAQP4 IgG–treated rats. In this regard, we collected the CSF sample of our rat models from the cisterna magna. However, blood contamination due to the hemorrhage caused by laminectomy while preparing the injection model was unavoidable. Thus, the comparison of ATP released into the CSF was not able to be compared between the two groups, confounded by the artificial contamination of blood contents (data not shown). This issue is also a limitation of this research.

## Conclusion

We showed that AQP4-Ab promotes extracellular ATP release from astrocytes, and ATP-mediated neuropathic pain was suggested in NMOSD rat model. Our results indicate the critical role of ATP in the pathogenesis of AQP4-Ab–mediated pain development.

## Supplementary Information

**Additional file 1: Supplementary Figure 1.** Recombinant AQP4 IgG binds to rat primary astrocytes with target antigen confirmed to be AQP4 by an adsorption assay.

**Additional file 2: Supplementary Figure 2.** Thermal allodynia was not induced by recombinant AQP4 IgG.

**Additional file 3: Supplementary Figure 3.** Intraspinal injection of anti-AQP4 antibody positive NMOSD serum induces mechanical allodynia.

**Additional file 4: Supplementary Figure 4.** The ATP release from HEK-AQP4 with rAQP4 IgG was cancelled by heat deactivation of human serum.

**Additional file 5: Supplementary Figure 5.** IL-1β and IL-6 concentration of CSF derived from NMOSD patients were not elevated.

**Additional file 6: Supplementary Table 1.** Gene list of Fold change>2.0 and P-Value<0.05

## Data Availability

The data that support the findings of this study are available from corresponding author on reasonable request.

## Abbreviations

AQP4-Ab: Anti-AQP4 recombinant autoantibodies
DAMPs: Damage-associated molecular patterns
HMGB1: High mobility group box-1
DEG: Differentially expressed gene
HEK293: Human Embryonic Kidney cells 293
HEK-AQP4: HEK293 cells expressing AQP4
MS: Multiple sclerosis
NMOSD: Neuromyelitis optica spectrum disorder
OND: Other neurological disease
PCA: Principal component analysis
rAQP4 IgG: Recombinant monoclonal AQP4 antibody
VAS: Visual analog scale
