# Supplementary material for: Anti-AQP4 autoantibodies promote ATP release from astrocytes and induce mechanical pain in rats
Source: J Neuroinflammation. 2021 Aug 21;18:181. doi: 10.1186/s12974-021-02232-w (PMC8380350; doi:10.1186/s12974-021-02232-w)
Supplement: Supplementary file 1 — Additional file 1: Supplementary Figure 1. Recombinant AQP4 IgG binds to rat primary astrocytes with target antigen confirmed to be AQP4 by an adsorption assay. [file 12974_2021_2232_MOESM1_ESM.docx]

**
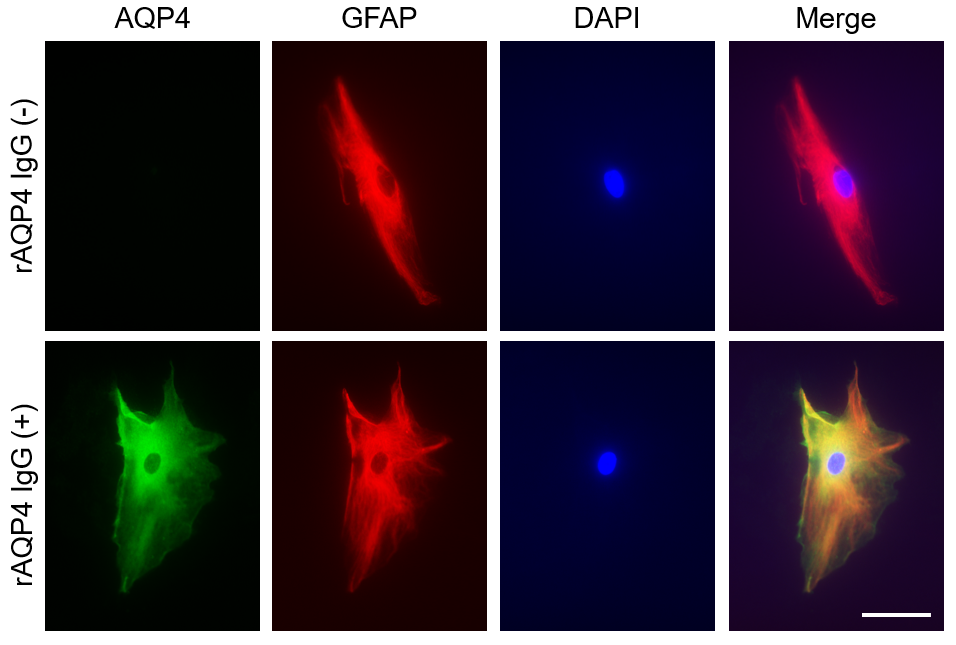
**

**A**

**
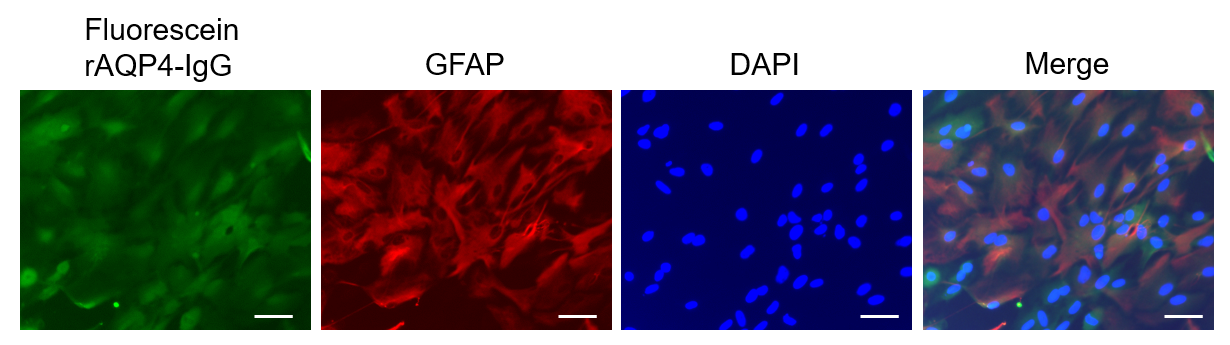
**

**B**

**C**


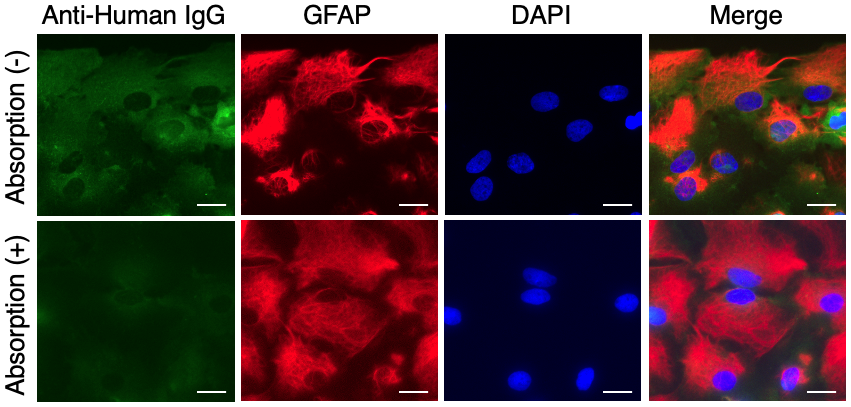


**Supplementary Figure 1**

**Recombinant AQP4 IgG binds to rat primary astrocytes with target antigen confirmed to be AQP4 by an adsorption assay.**

(A) Rat primary astrocytes were fixed in 4% paraformaldehyde for 15 min. After incubation with 1% BSA in PBS for 30 minutes at room temperature, rat astrocytes were incubated with or without recombinant AQP4 IgG (1:50) overnight at 4℃. Rat astrocytes were incubated with goat anti-human IgG FITC (1:50; Abcam, Cambridge, UK) for 30 minutes at room temperature. Subsequently rat astrocytes were incubated with GFAP mouse monoclonal antibody (Alexa Fluor 594 Conjugate) (1:50; Cell Signaling Technology, Beverly, MA, USA) for 30 minutes at room temperature. Scale bar = 25 µm.

(B) Recombinant AQP4 IgG was directly labelled by the Fluorescein Labeling Kit-NH2 (Dojindo, Kumamoto, Japan). Rat primary astrocytes were fixed in 4% paraformaldehyde for 15 min. After incubation with 1% BSA in PBS for 30 minutes at room temperature, rat astrocytes were incubated with rAQP4 IgG labelled by the fluorescein overnight at 4℃. Rat astrocytes were subsequently incubated with anti-GFAP mouse monoclonal antibody (Alexa Fluor 594 Conjugate) (1:50; Cell Signaling Technology, Beverly, MA, USA) for 30 minutes at room temperature. Scale bar = 25 µm.

(C) Absorption of rAQP4 IgG with AQP4 transfected HEK293 cells (HEK-AQP4). The rAQP4 IgG were diluted with 0.2% BSA-PBS to a final concentration of 0.2mg/ml IgG and added to HEK-AQP4 or HEK293 cells. After an overnight incubation, rAQP4 IgG were collected from each condition, thus generating the samples with or without AQP4 absorption. Rat primary astrocytes were fixed in 4% paraformaldehyde for 15 min. After incubation with 1% BSA in PBS for 30 minutes at room temperature, rat astrocytes were incubated with rAQP4 IgG with or without absorption overnight at 4℃. Rat astrocytes were subsequently incubated with goat anti-human IgG FITC (1:50; Abcam, Cambridge, UK) for 30 minutes at room temperature. Rat astrocytes were then incubated with anti-GFAP mouse monoclonal antibody (Alexa Fluor 594 Conjugate) (1:50; Cell Signaling Technology, Beverly, MA, USA) for 30 minutes at room temperature. Scale bar = 10 µm
