# Supplementary material for: Anti-AQP4 autoantibodies promote ATP release from astrocytes and induce mechanical pain in rats
Source: J Neuroinflammation. 2021 Aug 21;18:181. doi: 10.1186/s12974-021-02232-w (PMC8380350; doi:10.1186/s12974-021-02232-w)
Supplement: Supplementary file 2 — Additional file 2: Supplementary Figure 2. Thermal allodynia was not induced by recombinant AQP4 IgG. [file 12974_2021_2232_MOESM2_ESM.docx]

**
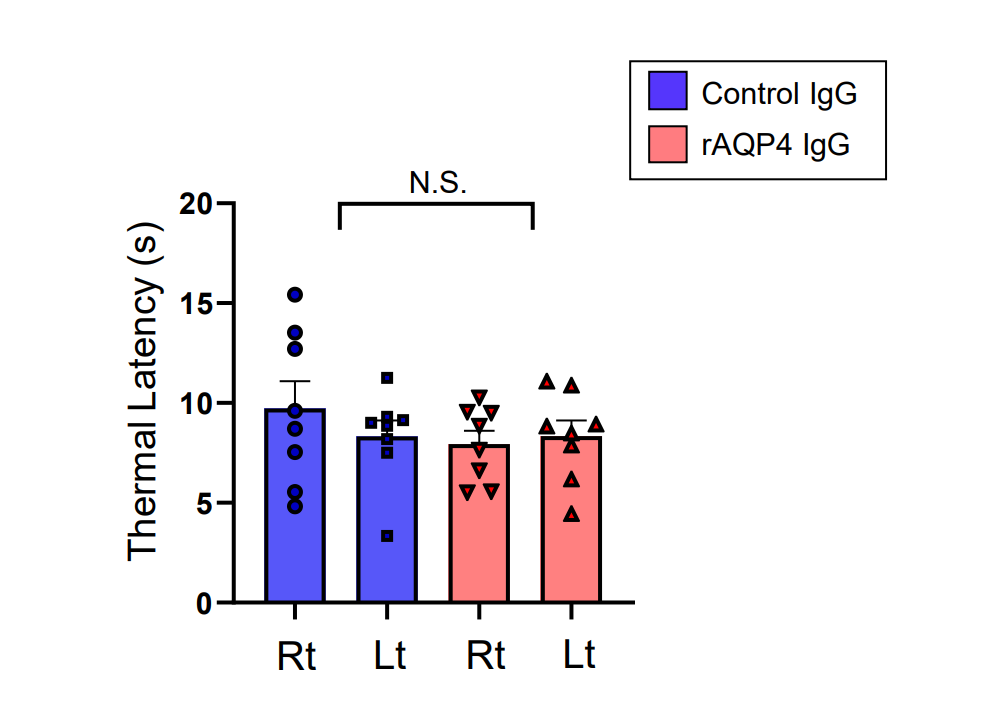
**

**Supplementary Figure 2**

**Thermal allodynia was not induced by recombinant AQP4 IgG.**

Heat hyperalgesia was assessed using a Hargreaves radiant heat apparatus (Ugo Basile, Gemonio, Italy). The heat source, a mobile infrared photobeam, was positioned under the plantar surface of the hind paw. The intensity of the heat stimulus was adjusted to 50, and the cutoff was set to 20 s. Thresholds of thermal allodynia were assessed 3 days after intraspinal injection of either control IgG (n = 8) or recombinant aquaporin-4 antibody (rAQP4 IgG) (n = 8) with normal human serum containing complement. Data are expressed as means ± SEM, and were analyzed by one-way repeated measures ANOVA with one within-subjects factor [the same rat’s paired right (Rt) and left (Lt) data]. N.S., not significant.
