# Supplementary material for: Anti-AQP4 autoantibodies promote ATP release from astrocytes and induce mechanical pain in rats
Source: J Neuroinflammation. 2021 Aug 21;18:181. doi: 10.1186/s12974-021-02232-w (PMC8380350; doi:10.1186/s12974-021-02232-w)
Supplement: Supplementary file 3 — Additional file 3: Supplementary Figure 3. Intraspinal injection of anti-AQP4 antibody positive NMOSD serum induces mechanical allodynia. [file 12974_2021_2232_MOESM3_ESM.docx]

**
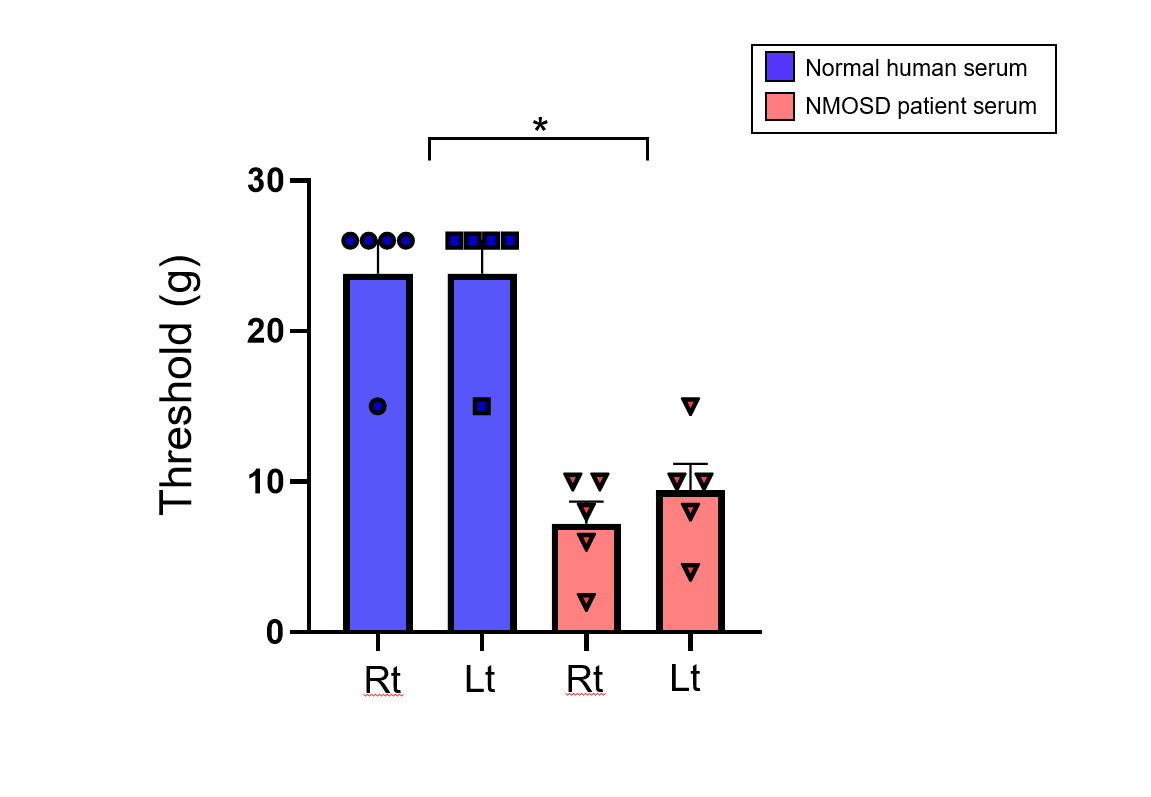
**

**Supplementary Figure 3**

**Intraspinal injection of anti-AQP4 antibody positive NMOSD serum** **induces mechanical allodynia.**

Thresholds of mechanical allodynia assessed 3 days after intraspinal injection of either 3μl of normal human serum (n = 5) or anti-AQP4 antibody positive NMOSD patient serum (n = 5). Data are expressed as means ± SEM, and were analyzed by one-way repeated measures ANOVA with one within-subjects factor [the same rat’s paired right (Rt) and left (Lt) data]. * P < 0.05
