# Supplementary material for: Anti-AQP4 autoantibodies promote ATP release from astrocytes and induce mechanical pain in rats
Source: J Neuroinflammation. 2021 Aug 21;18:181. doi: 10.1186/s12974-021-02232-w (PMC8380350; doi:10.1186/s12974-021-02232-w)
Supplement: Supplementary file 4 — Additional file 4: Supplementary Figure 4. The ATP release from HEK-AQP4 with rAQP4 IgG was cancelled by heat deactivation of human serum. [file 12974_2021_2232_MOESM4_ESM.docx]

**
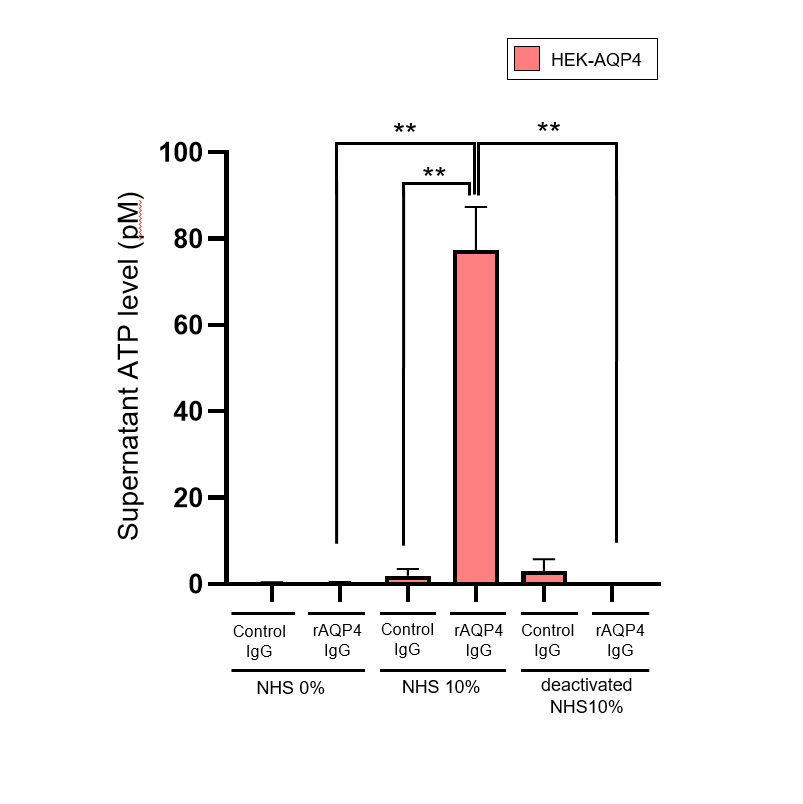
**

**Supplementary Figure 4**

**The ATP release from HEK-AQP4 with rAQP4 IgG was cancelled by heat deactivation of human serum.**

The extracellular ATP level in culture medium was lower in rAQP4 IgG with 10% heat deactivated human serum group than in rAQP4 IgG with 10% non-deactivated human serum group. The deactivated human serum was transferred to fresh siliconized tubes and immersed in a 56°C water bath for 30 min before experiment. Data are expressed as means ± SEM, and were analyzed by factorial ANOVA with three between-subjects factors [Deactivation: deactivation and non-deactivation, Group: rAQP4 and control, IgG level: 0% and 10%]. The main effects of Deactivation (P<0.001), Group (P<0.001) and IgG (P<0.001) and the interactions of Deactivation times Group (P<0.001) and Deactivation times IgG (P<0.001) were statistically significant. For the visualization, the asterisks indicate three two-group comparisons, **P < 0.01.
