# Supplementary material for: Anti-AQP4 autoantibodies promote ATP release from astrocytes and induce mechanical pain in rats
Source: J Neuroinflammation. 2021 Aug 21;18:181. doi: 10.1186/s12974-021-02232-w (PMC8380350; doi:10.1186/s12974-021-02232-w)
Supplement: Supplementary file 5 — Additional file 5: Supplementary Figure 5. IL-1β and IL-6 concentration of CSF derived from NMOSD patients were not elevated. [file 12974_2021_2232_MOESM5_ESM.docx]

**
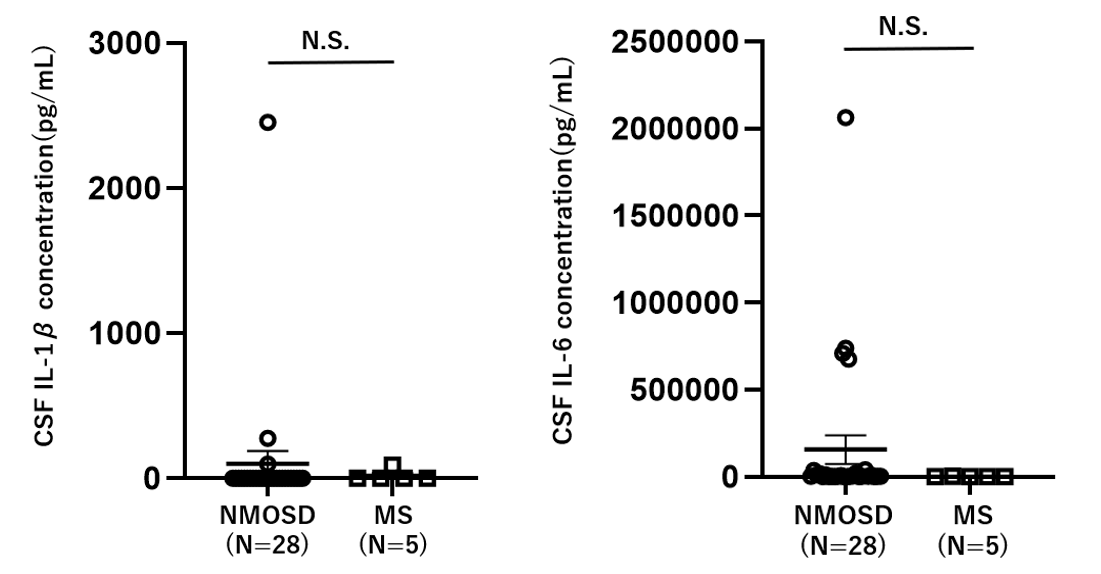
**

**Supplementary Figure 5**

**IL-1β and IL-6 concentration of CSF derived from NMOSD patients were not elevated.**

IL-1β and IL-6 concentration of CSF derived from NMOSD and MS patients were analyzed using BD™ Cytometric Bead Array assays (BD-bioscience, San Jose, CA, USA). Data are expressed as means ± SEM, and were analyzed by Student’s t-test. N.S., not significant.
